# SLPW: A Virulent Bacteriophage Targeting Methicillin-Resistant *Staphylococcus aureus In vitro* and *In vivo*

**DOI:** 10.3389/fmicb.2016.00934

**Published:** 2016-06-15

**Authors:** Zhaofei Wang, Panpan Zheng, Wenhui Ji, Qiang Fu, Hengan Wang, Yaxian Yan, Jianhe Sun

**Affiliations:** Shanghai Key Laboratory of Veterinary Biotechnology, Key Laboratory of Urban Agriculture (South), Ministry of Agriculture, School of Agriculture and Biology, Shanghai Jiao Tong UniversityShanghai, China

**Keywords:** *Staphylococcus aureus*, MRSA, phage SLPW, infection, therapy

## Abstract

*Staphylococcus aureus* (*S. aureus*) is a Gram-positive pathogen causing a variety of infections in humans and animals. Extensive use of antibiotics has led to the emergence of methicillin-resistant *S. aureus* (MRSA). As an alternative antibacterial agent against drug-resistant *S. aureus*, a lytic phage, designated SLPW, was isolated from fecal sewage in a pig farm. The SLPW was morphologically classified under *Podoviridae* and contains a double-stranded DNA genome. The genome of SLPW was 17,861 bp (29.35% G+C) containing 20 open reading frames and lacked regions encoding lysogeny-related integrase gene and *cI* repressor gene. Phage SLPW showed a broad host range and high efficiency of plating against various types of *S. aureus*. One-step growth curve showed a short latency period (10 min) and a long lytic period (120 min). Phage SLPW remained stable under a wide range of temperatures or pH and was almost unaffected in chloroform or ultraviolet light. Further, it efficiently lysed MRSA strains *in vitro* and *in vivo*. Intraperitoneal phage administration at 1 h post-infection cured the mice and reduced the bacterial expression of inflammatory cytokines in mice. Specifically, the phage SLPW displayed a wide antibacterial spectrum. It was therapeutically effective against intra-abdominal infection in mice harboring different multilocus sequence typing (MLST) types of *S. aureus* strains. Therefore, phage SLPW is a potential therapeutic agent against MRSA infections.

## Introduction

*Staphylococcus aureus* (*S. aureus*) is the most virulent pathogen causing various diseases, including skin abscesses, pneumonia, endocarditis, and osteomyelitis, in humans and animals (Lowy, 1998; Plata et al., 2009). The two major sources of infection include community and hospital (Engemann et al., 2003). The bacterial strains are resistant to many antibiotics, and especially to methicillin and vancomycin. The emergence and prevalence of methicillin-resistant *S. aureus* (MRSA) and vancomycin-resistant *S. aureus* (VRSA) underscores the need for development of effective therapeutic alternatives (Sasidharan et al., 2011; Gardete and Tomasz, 2014).

In the last 15 years, there has been a marked increase in the number of identified *Staphylococcus* phages, with tremendous progress in therapeutic interventions targeting *Staphylococcus*, especially *S. aureus* (Hsieh et al., 2011). Phages are the most common organisms on the planet and represent great diversity in host range. *S. aureus* phages target pathogens in diseases, such as bacteremia, eye infections, and *S. aureus-*associated lung infections (Wills et al., 2005; Kazmierczak et al., 2015). Compared with traditional antibiotics, bacteriophages are cost-effective without serious side effects, and are virulent especially against drug-resistant bacteria (Borysowski et al., 2011; Kazmierczak et al., 2015). Further, phages generally recognize specific receptors on bacterial cell membrane, without affecting human, or animal cells. Therefore, the side effects in eukaryotic hosts are minimal (Sulakvelidze et al., 2001). Studies involving *S. aureus* phages show effective and comprehensive antimicrobial activity *in vitro* and *in vivo* (Capparelli et al., 2007; Gutierrez et al., 2015).

In this study, we isolated a lytic phage, SLPW, from fecal sewage in a pig farm. We report the wide host range, adequate stability and strong bacteriolytic activity of this phage. Specifically, the phage SLPW was safe and effective against MRSA infection in mice.

## Materials and methods

### Ethics statement

Animal experiments were carried out according to the animal welfare standards approved by the Ethical Committee for Animal Experiments of Shanghai Jiao Tong University, China. All animal experiments complied with the guidelines of the Animal Welfare Council of China.

### Bacterial strains and culture conditions

In this study, 38 *S. aureus* strains (18 MRSA strains, 7 clinically isolated pathogenic strains and 13 strains isolated from milk samples of dairy cows with mastitis) and 8 other strains (*Staphylococcus epidermidis* ATCC12228, *Bacillus subtilis* YS, *S. zooepidemicus* ATCC35246, 4 *Streptococcus suis* and *Escherichia coli* MC1061) were used (Table 1). Two reference strains of *S. aureus* ATCC 25923 and ATCC 29213 from the American Type Culture Collection (ATCC) were also used. All the strains were grown in Todd–Hewitt broth (THB) and brain heart infusion (BHI) or agar medium supplemented with 2% (vol/vol) fetal bovine serum (Gibco, Invitrogen Corp., Carlsbad, CA) at 37°C.

**Table 1:** **SLPW strains and lytic activity**.

**Species of strain**	**No. of strain**	**MLST type**	**EOP^a^**	**Source^b^**
*Staphylococcus aureus* (22)	ATCC25923	ST 243	1	III
	ATCC29213	ST 5	1.37 × 10^−5^	III
	S1	ST 398	4.24 × 10^−6^	I
	S2	ST 239	6.12 × 10^−1^	I
	S3	ST 239	—	I
	S4	ST 239	2.13 × 10^−1^	I
	S5	ST 398	6.33 × 10^−1^	I
	S6	ST 9	—	I
	S7	ST 239	8.22 × 10^−5^	I
	SH-5	ST 9	2.32 × 10^−1^	II
	SH-6	ST 9	5.44 × 10^−7^	II
	SH-7	ST 9	7.21 × 10^−6^	II
	SH-8	ST 9	8.14 × 10^−1^	II
	SH-9	ST 9	2.02 × 10^−1^	II
	SH-10	ST 9	4.37 × 10^−1^	II
	SH-11	ST 9	2.62 × 10^−1^	II
	SH-12	ST 9	4.23 × 10^−1^	II
	SH-13	ST 9	4.22 × 10^−5^	II
	SH-14	ST 9	9.17 × 10^−6^	II
	SH-15	ST 9	1.98 × 10^−1^	II
	SH-16	ST 9	2.82 × 10^−6^	II
Methicillin-resistant *Staphylococcus aureus* (18)	SH-17	ST 9	1.81 × 10^−1^	II
	MS3	ST 9	1.62	IV
	MS5	ST 5	7.52 × 10^−1^	IV
	MS6	ST 5	2.88 × 10^−1^	IV
	MS7	ST 5	7.23 × 10^−1^	IV
	MS8	ST 9	8.22 × 10^−1^	IV
	MS9	ST 9	8.21 × 10^−1^	IV
	MS10	ST 239	2.82 × 10^−6^	IV
	MS11	ST 9	4.33 × 10^−1^	IV
	MS13	ST 9	8.22 × 10^−1^	IV
	MS15	ST 5	8.17 × 10^−1^	IV
	MS16	ST 9	9.23 × 10^−1^	IV
	MS17	ST 398	4.33 × 10^−5^	IV
	MS18	ST 9	3.22 × 10^−1^	IV
	MS19	ST 9	2.98 × 10^−1^	IV
	MS20	ST 398	1.23 × 10^−1^	IV
	MS21	ST 398	1.67 × 10^−1^	IV
	MS22	ST 398	—	IV
	MS23	ST 9	—	IV
*Staphylococcus epidermidis*	ATCC12228	—^c^	—	IV
*Bacillus subtilis*	YS	—	—	IV
*Streptococcus suis*	SS1	—	—	IV
	SS2	—	—	IV
	SS7	—	—	IV
	SS9	—	—	IV
*Streptococcus zooepidemicus*	ATCC35246	—	—	III
*Escherichia coli*	MC1061	—	—	IV

a EOP, efficiency of plating (EOP = phage titer on test bacterium / phage titer on host bacterium Staphylococcus aureus ATCC25923). Assays were conducted at least three times. The data shown represent means derived from three independent experiments.

b I, clinically-isolated pathogenic strains; II, isolated from milk samples of dairy cows with mastitis; III, purchased from American Type Culture Collection; IV, stored in our lab.

c —, no plaque on target bacterium.

### Phage isolation, purification, and host range determination

The method of Matsushiro et al. was adopted for the isolation of *S. aureus* phages with some modifications (Matsushiro and Okubo, 1972). Seventy-four samples, including 44 dust swabs and 30 fecal samples were suspended in SM buffer [NaCl 5.8 g/L, MgSO_4_ · 7H_2_O 2 g/L, 1 M Tris·HCl (pH7.5) 50 ml/L, and 2% gelatin 5 ml/L] and centrifuged at 5000 × *g* for 20 min at 4°C. The supernatants were filtered through 0.22-μm pore membranes and evaluated for the presence of lytic phages using different *S. aureus* isolates on BHI plates. After overnight incubation, bacterial plaque formation suggested the presence of lytic phage, which was purified after three rounds of single-plaque isolation.

For purification, a single-phage plaque was precipitated in the presence of 10% (wt/vol) polyethylene glycol (PEG) 8000 and 1 M NaCl at 4°C for at least 1 h. The precipitate was collected by centrifugation at 10,000 × *g* for 10 min at 4°C and suspended in SM buffer. After the addition of 0.5 g/mL CsCl, the mixture was layered on top of CsCl step gradients (densities of 1.15, 1.45, 1.50, and 1.70 g/mL) in Ultra-Clear centrifugation tubes and centrifuged at 28,000 × *g* for 2 h at 4°C, and dialyzed in sodium chloride–magnesium sulfate buffer [100 mM NaCl, 10 mM MgSO_4_ · 7H_2_O, and 50 mM Tris·HCl (pH 7.5)]. Phages were stored at 4°C for further experiments.

The host range of the phage was defined by the double-layered agar method described by Adams (1959). The SLPW phage was inoculated with all the 47 strains listed in Table 1 and then monitored for plaque formation.

### Transmission electron microscopy (TEM) of phage particles

The purified phage sample was loaded onto a copper grid for 7 min followed by negative staining with 2% (vol/vol) uranyl acetate (pH 6.7) and drying. The phage morphology was observed using a FEI TEM Tecnai G2 Spirit Biotwin (FEI, Hillsboro, US) at an accelerating voltage of 80 kV.

### Restriction enzyme digestion of phage genomic DNA

Purified phage genomic DNA was prepared as described previously by Son et al. (2010). For the identification of nucleic acid type, purified phage genomic DNA was subjected to nuclease treatment using D*Nase* I (20 U/μL), R*Nase* A (5 U/μL), and Mung bean nuclease (20 U/μL) at 37°C for 1 h. Restriction site analysis of the phage was conducted by digesting purified phage genomic DNA with 10 U *Xho* I, *EcoR* I, *Hind* III, *Ned* I, and *Not* I for 1 h at 37°C. Products of digested phage nucleic acid were separated by 0.8% (wt/vol) agarose gel electrophoresis.

### Genome sequencing and annotation

Shotgun sequencing was used for Phage SLPW whole genome analysis. Sequence alignments were carried out using the Accelrys DS Gene software package of Accelrys Inc. (USA). Putative open reading frames were suggested using the algorithms of the software packages Accelrys Gene v2.5 (Accelrys Inc.) and ORF Finder (NCBI). Identity values were calculated using different BLAST algorithms (http://www.ncbi.nlm.nih.gov/BLAST/) at the NCBI homepage. The sequence of phage SLPW has been submitted to NCBI (GenBank accession number: KU992911).

### Assay of optimal multiplicity of infection (MOI)

Overnight cultures of *S. aureus* ATCC 25923 strain were diluted 1:100 in fresh BHI and incubated at 37°C with shaking until early logarithmic growth phase (optical density at 600 nm, 0.4–0.6), diluted 1:10, and mixed with phages at different MOIs. After 3.5 h incubation at 37°C, the mixture was centrifuged at 5000 × *g* for 20 min at 4°C and the supernatants were filtered through 0.22-μmporesize membranes. The phage titer in the supernatant was immediately determined using a double-layer agar plate method. This assay was performed at least in triplicate.

### One-step growth

For determination of one-step growth of phage SLPW, we used *S. aureus* ATCC 25923 as the host strain because it displayed the largest clearance zone in a spot test and was lysed rapidly to yield a clear lysate in liquid culture. One-step growth experiments were performed using a modified method described previously (Pajunen et al., 2000). Briefly, SLPW phage was added at a MOI of 0.1 to the cells of *S. aureus* and allowed to adsorb for 15 min at 37°C. The mixture was then centrifuged at 10,000 × *g* for 1 min. After the supernatants were removed, the pellets containing the phage-infected bacterial cells were suspended in fresh BHI and incubated with shaking at 180 rpm and 37°C. Partial samples were obtained at 10 min intervals and the titrations from the aliquots were immediately determined using the double-layer agar plate method. This assay was performed at least in triplicate.

### Phage stability

Phage stability was determined at different temperatures (25, 37, 45, 50, 55, 60, 65, and 70°C), using an aliquot of phage SLPW obtained after 1 h. The titers of the phage lysate were assayed using a double-layer agar plate method. The phage stability at different pH-values was tested by determining the titers after dilution of the phage lysates (1:100) in SM buffer and stored at 37°C for 3 h. To analyze the chemical stability, the phage SLPW was treated with chloroform (5, 25, 50, or 75%, vol/vol) for 6, 12, 18, and 24 h at 4°C. In addition, the phage SLPW was also exposed to ultraviolet light treatment for 10, 20, 30, 40, 50, and 60 min, and titrated immediately using a double-layer agar plate method.

### Efficiency of plating

Phage SLPW was screened against *S. aureus* strains using the efficiency of plating method (EOP = phage titer on test bacterium/phage titer on host bacterium) to determine the effectiveness against a variety of target bacteria. Ten-fold serial dilutions of phage suspensions (100 μL) were mixed with 100 μL of the target or host bacterium (grown overnight at 37°C) and incubated for 5 min at room temperature (25°C) and plated as double layers on THB (Viscardi et al., 2008).

### Phage bacteriolytic activity *In vitro*

Overnight cultures of *S. aureus* culture were diluted 1:100 in fresh THB liquid medium incubated at 37°C with shaking at 180 rpm until an early-exponential host bacterial culture (optical density at 600 nm, 0.4–0.6) was reached. Phage SLPW was added at MOI of 0.01, 1, and 100, and an identical *S. aureus* culture with the same volume phage diluent was used as the control. The mixture was then grown at 37°C with shaking at 180 rpm. The phage bacteriolytic activity was assessed by monitoring the cell absorbance of the culture solution (OD_600_) at 30-min intervals for up to 4 h, and this assay was performed in triplicate.

### Phage protection studies

Female BALB/c mice (6 weeks of age) were purchased from the Experimental Animal Center, Shanghai Jiao Tong University. Overnight cultures of *S. aureus* were diluted 1:100 in fresh THB liquid medium incubated at 37°C with shaking at 180 rpm to an early-exponential host bacterial culture (optical density at 600 nm, 0.4–0.6). Cells were pelleted and washed twice with phosphate-buffered saline (PBS). The mice were infected with a dose of 1 × 10^9^ CFU in 0.2 mL of the *S. aureus* strain. The bacterial cells were injected unilaterally into the abdominal cavities of mice, and 0.2 mL of the purified phage samples (1 × 10^9^ PFU) were injected into the other side immediately, at 60 and 120 min after a bacterial challenge. The controls included uninfected mice administered with 0.2 mL of phage in SM buffer. The mouse survival rate was recorded daily for 7 days. The CFU or PFU organ burden in spleen, lung, and blood was determined by sacrificing groups of six mice at 6, 12, and 24 h after phage SPLW administration. Each sample was homogenized in 1 mL PBS and serially diluted in PBS. CFU were evaluated by plating each dilution on THB agar plates. The PFU were evaluated by plating each dilution on the double-layer agar plate.

### Cytokine assays

To evaluate the antimicrobial effects of phage, 0.2 mL of phage in SM buffer (1 × 10^9^ PFU) was administered intraperitoneally at 1 h after infection with *S. aureus* (1 × 10^9^ CFU). SM buffer alone was administered to uninfected mice serving as control groups. Spleen tissues were removed from mice 6, 12, and 24 h after injection with phage SLPW. Tissues were homogenized in 1 mL of lysis buffer (Qiagen, West Sussex, UK), followed by centrifugation at 2000 × *g* for 10 min. The supernatants were sterilized with a millipore filter (0.45-μm pore size).

Total RNA was isolated from the supernatants using an AllPrep RNA microkit (Qiagen). The cDNA synthesis was performed using the PrimeScript RT reagent kit (TaKaRa, Dalian, China) according to the manufacturer's instructions. The mRNA levels were measured using two-step relative qRT-PCR. The β-actin housekeeping gene was amplified as an internal control. The sequences of the primers for tumor necrosis factor alpha (TNF-α), interleukin-6 (IL-6), interleukin-1β (IL-1β), and β-actin are listed in Table 2. Gene expression was normalized to the expression of the housekeeping gene β-actin. Real-time PCR was performed using a SYBR Premix Ex Taq kit (TaKaRa) and CFX Connect^*TM*^ RT-PCR system (BIO-RAD, Hercules, USA). The comparative cycle threshold (2^−ΔΔCT^) method was used to analyze the mRNA levels.

**Table 2:** **Primers used for qRT-PCR**.

**Primer**	**Sequence (5′–3′)**
IL-1β-F	TCCAGGATGAGGACATGAGCAC
IL-1β-R	GAACGTCACACACCAGCAGGTTA
IL-6-F	CCACTTCACAAGTCGGAGGCTTA
IL-6-R	GCAAGTGCATCATCGTTGTTCATAC
TNF-α-F	AAGCCTGTAGCCCACGTCGTA
TNF-α-R	GGCACCACTAGTTGGTTGTCTTTG
β-actin-F	TGACAGGATGCAGAAGGAGA
β-actin-R	GCTGGAAGGTGGACAGTGAG

### Statistical analyses

Experimental data points were plotted using GraphPad Prism 5 (GraphPad Software, Inc., San Diego, CA). Data were expressed as mean values ± standard errors of the means (SEM). The phage protection analyses were performed using the non-parametric Mann-Whitney U-test. A *P* < 0.05 was considered significant.

## Results and discussion

### Phage isolation and host range determination

In this study, we isolated a lytic *S. aureus* phage designated as SLPW, from fecal sewage in a pig farm of Shanghai (China) in 2013. Using *S. aureus* ATCC25923 as the host strain, the phage plaques measuring 1–2 mm in diameter were obtained. The phage SLPW had a strong ability to produce plaques on *S. aureus* strains. Among the 40 *S. aureus* strains, 36 (90%) isolates were lysed by SLPW (Table 1). Furthermore, the SLPW phage showed strong lytic activity against the majority of MRSA strains (16 of 18 strains), suggesting a potential therapeutic role in MRSA infection. However, no plaque production was observed in *Staphylococcus epidermidis, Bacillus subtilis, S. zooepidemicus, Streptococcus suis*, and *E. coli* strains investigated (Table 1).

**Figure 1:**
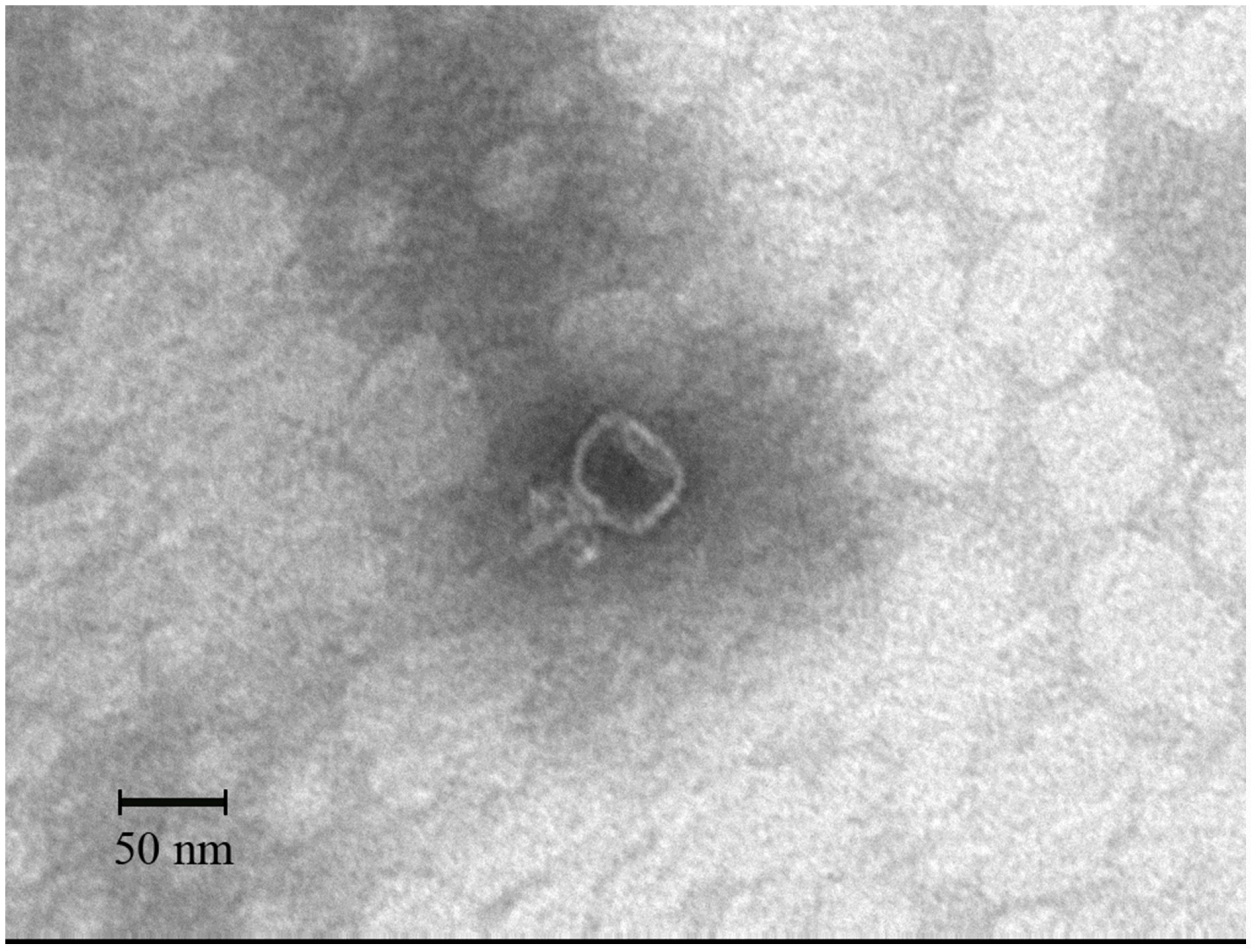
**Transmission electron microscopy of negatively-stained phage SLPW**.

The morphology of the isolated phage SLPW was determined. Electron microscopy showed that the SLPW particle had an isometric head of 49.5 ± 1.5 nm and a short, non-contractile tail measuring 19.5 ± 1.5 nm long (Figure 1). Thus, it was morphologically similar to phages of the family *Podoviridae* according to the classification of International Committee on Taxonomy of Viruses (ICTV; Adams et al., 2014).

### Phage nucleic acid type and genome description

**Figure 2:**
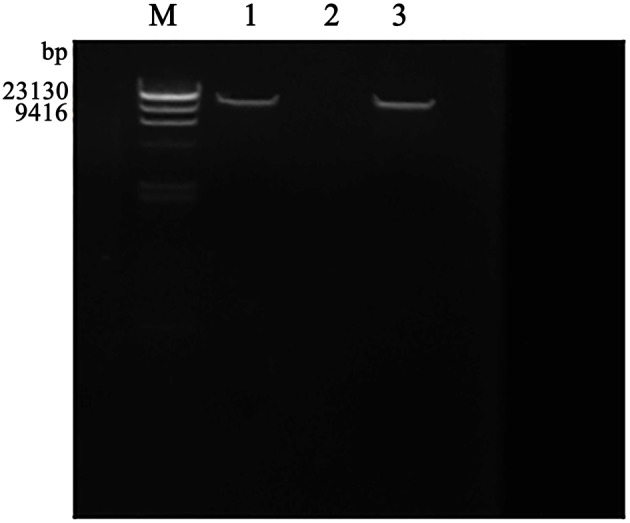
**Agarose gel electrophoresis of phage SLPW genome digested with nuclease**. Lane M: λ-*Hind* III digest DNA Marker, Lane 1–3: phage SLPW genome digested with R*Nase*A, D*Nase* I, and Mung Bean Nuclease, respectively.

**Figure 3:**
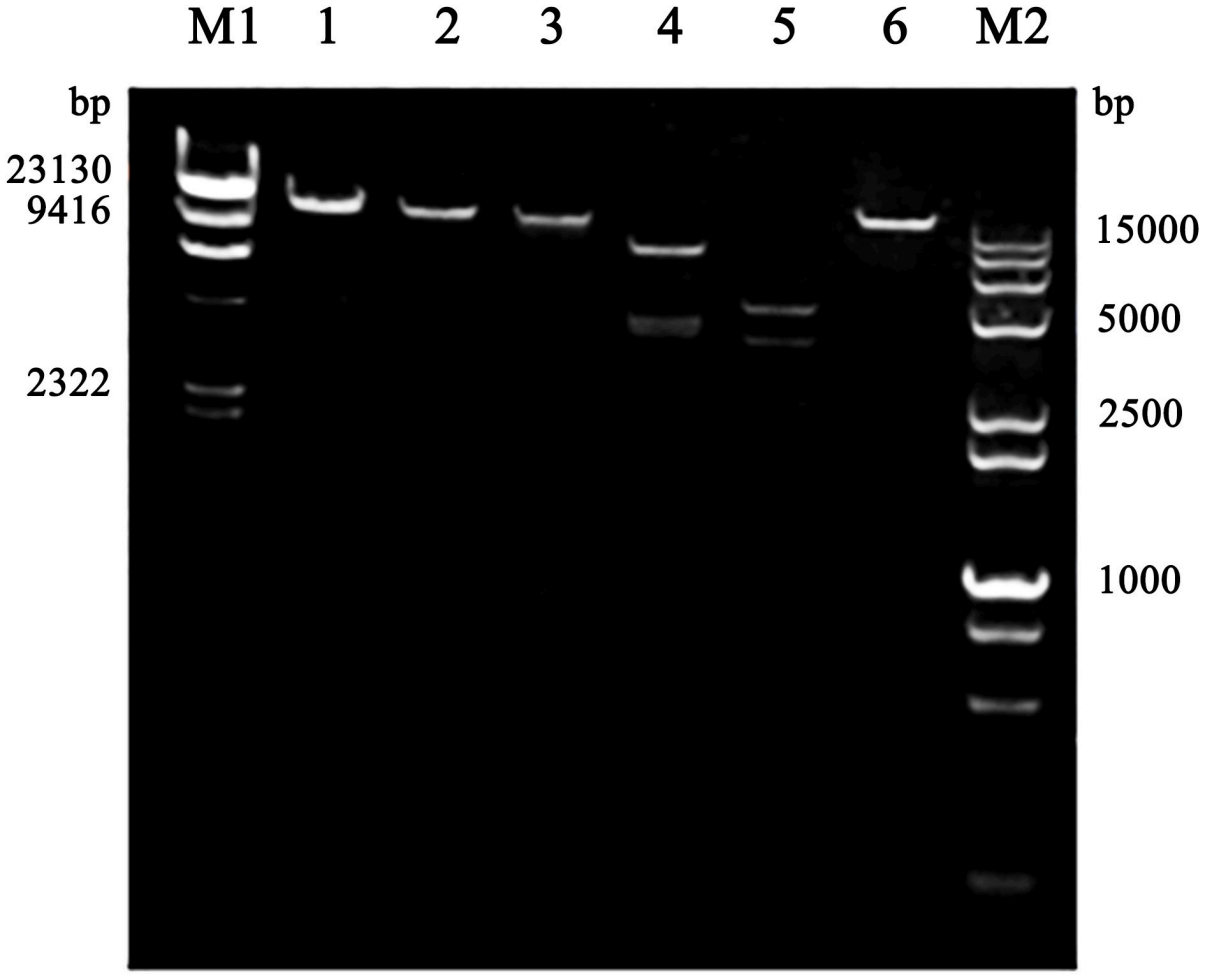
**Restriction digestion patterns of phage SLPW DNA**. Lane M1: λ-*Hind* III digested DNA Marker, Lane 1: phage SLPW genome, Line 2–6: phage SLPW genome digested with *Xho* I*, EcoR* I*, Hind* III*, Ned* I, and *Not* I, respectively, Line M2: DL 15000+2000 marker.

The purified phage genomic DNA was subjected to digestion by different nucleases. The results showed that the genome of phage SLPW was completely digested by D*Nase* I but not by R*Nase* A or Mung bean nuclease (Figure 2), suggesting that phage SLPW was a double-stranded DNA. Purified phage SLPW genomic DNA could be digested with several restriction endonucleases including *Xho* I, *EcoR* I, *Hind* III, and *Ned* I (Figure 3).

The complete nucleotide sequence of phage SLPW was determined. The SLPW genome comprises 17,861 bp with an average G+C content of 29.35%, which is similar to that of the lytic *Staphylococcus* phages S13', PSa3, and 66 (Table S1). As shown in Table S2, 20 open reading frames (ORFs) were defined as potential genes of SLPW. Genes involved in packaging, head, tail, lysis, and DNA replication showed high homology with other phages listed in Figure S1 and Table S2 (Kwan et al., 2005). However, a few unknown proteins of SLPW including Gp4, Gp6, and Gp20 showed a lower degree of similarity than those of other *Podoviridae Staphylococcus* phages (Table S2), which may lead to functional discrepancy. The genes of SLPW encoding lysin (Gp14) and holin (Gp10) showed high similarity with *Staphylococcus* lytic phages listed in Table S2, which also showed a broad host range and strong lytic ability against *S. aureus* (Kraushaar et al., 2013). Comparison of the genome structure with *Staphylococcus* prophages suggested that SLPW was a lytic phage, which lacked specific integration-related and *cI* repressor genes, devoid of lysogenic characteristics (Kwan et al., 2005; Hoshiba et al., 2010; Biswas et al., 2014).

### Determination of optimal multiplicity of infection (MOI) and one-step growth curve

**Figure 4:**
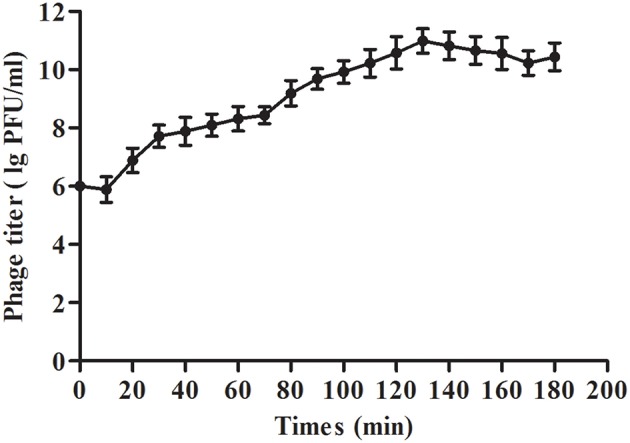
**One-step growth curve of phage SLPW in *S. aureus***. Phage SLPW was co-incubated with *S. aureus* ATCC25923 strain cultured at an MOI of 0.1 for 15 min at 37°C. The mixture was centrifuged to remove non-absorbed phage. The re-suspended pellets were incubated at 37°C and sampled at 10 min intervals over a period of 3 h. Phage titer was measured. Results are shown as means ± SEM from triplicate experiments. The latent period was 10 min: interval between the absorption and the beginning of the initial burst. The burst size was estimated at 95.3 PFU per infected cell, which was the ratio of the final count of liberated phage particles to the initial count of infected bacterial cells.

The results showed that the optimal MOI of phage SLPW was 0.1, which was the highest titer attained by the phage lysates (4.87 × 10^10^ PFU/mL; Table 3). Based on the optimal MOI, we established a one-step growth curve. Short latency period (10 min) and large burst size (estimated at 95.3 PFU per infected cell; Figure 4) suggest lytic nature of the SLPW phage and a higher lytic activity than the previously published lytic *S. aureus* phages (Han et al., 2013; Li and Zhang, 2014).

**Table 3:** **Optimal multiplicity of infection (MOI) of phage SLPW**.

**CFU of *S. aureus* ATCC25923 strain**	**PFU of phage SLPW**	**MOI**	**Phage SLPW titers (PFU/mL)**
10^6^	10^8^	100	3.32 × 10^9^
10^7^	10^8^	10	2.71 × 10^9^
10^8^	10^8^	1	3.12 × 10^10^
10^8^	10^7^	0.1	4.87 × 10^10^
10^8^	10^6^	0.01	2.92 × 10^8^
10^8^	10^5^	0.001	4.41 × 10^7^
10^8^	10^4^	0.0001	3.37 × 10^6^

### Phage stability

**Figure 5:**
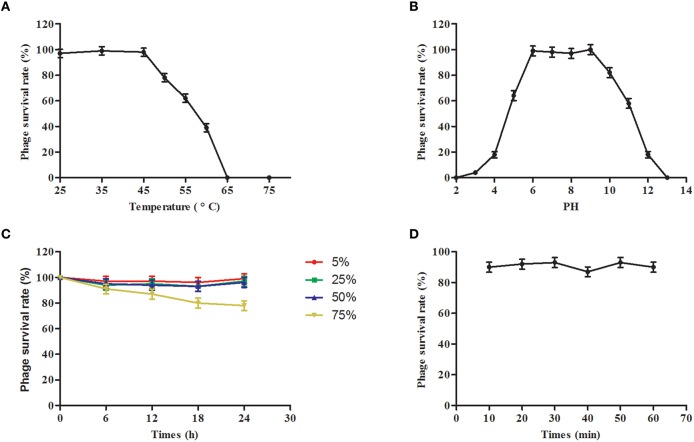
**Stability tests of phage SLPW**. **(A)** Thermostability: Phage SLPW was incubated at various temperatures as indicated. Samples were collected after 1 h; **(B)** pH stability: Phage SLPW was incubated under different pH conditions for 3 h; **(C)** Chloroform stability: Phage SLPW was treated with chloroform (5, 25, 50, or 75%, vol/vol) for 6, 12, 18, and 24 h; **(D)** Ultraviolet light stability: Phage SLPW was exposed to UV light for 10, 20, 30, 40, 50, and 60 min. The overall results were expressed as survival rates, and were titrated immediately using double-layer agar plate method. Results are shown as means ± SEM from triplicate experiments.

The potential clinical role of phage SLPW was evaluated by determining their physical and chemical stabilities. The thermal stability of the phages was investigated at different temperatures. We found that the activity of phage SLPW was stable at temperatures up to 45°C. Higher temperatures resulted in progressive inactivation. Phage SLPW was completely inactivated when heated to 65°C (Figure 5A). The pH stability was studied in SM buffers at a pH range of 2–12. Phage SLPW showed a relatively high survival rate (more than 80%) at a pH ranging from 6 to 10. Beyond these values, the activity decreased dramatically (Figure 5B). Further, the viability of phage SLPW was almost unaffected in the presence of 5, 25, 50, and 75% chloroform as shown in Figure 5C. Ultraviolet irradiation assay showed that about 90% of phage SLPW survived UV light (30 w, 30 cm wave-length) treatment ranging from 10 to 60 min (Figure 5D).

Studies suggested a probable relationship between phage structure and survival under adverse environmental conditions (Lasobras et al., 1997). Ackermann et al indicated that tailed phages remain comparatively steady in adverse conditions (Ackermann et al., 2004). Under harsh conditions, such as strong ultraviolet light, and large temperature fluctuations, phages belonging to *Myoviridae* protect themselves from extremely dry environment via intercellular location in pseudo-lysogens or biofilms created by bacterial hosts (Jonczyk et al., 2011). Phages from *Podoviridae* family may be extremely resistant to dry environment and survive large temperature fluctuations (Prigent et al., 2005). Our studies suggest that SLPW, which belongs to *Podoviridae*, showed a broad range of thermal and pH stability and strong resistance to chloroform and ultraviolet light treatment. Based on the above studies, tailed phages generally show great ability to adapt to adverse conditions, contributing to the development of phagotherapy.

### Bacteriolytic activity *In vitro*

**Figure 6:**
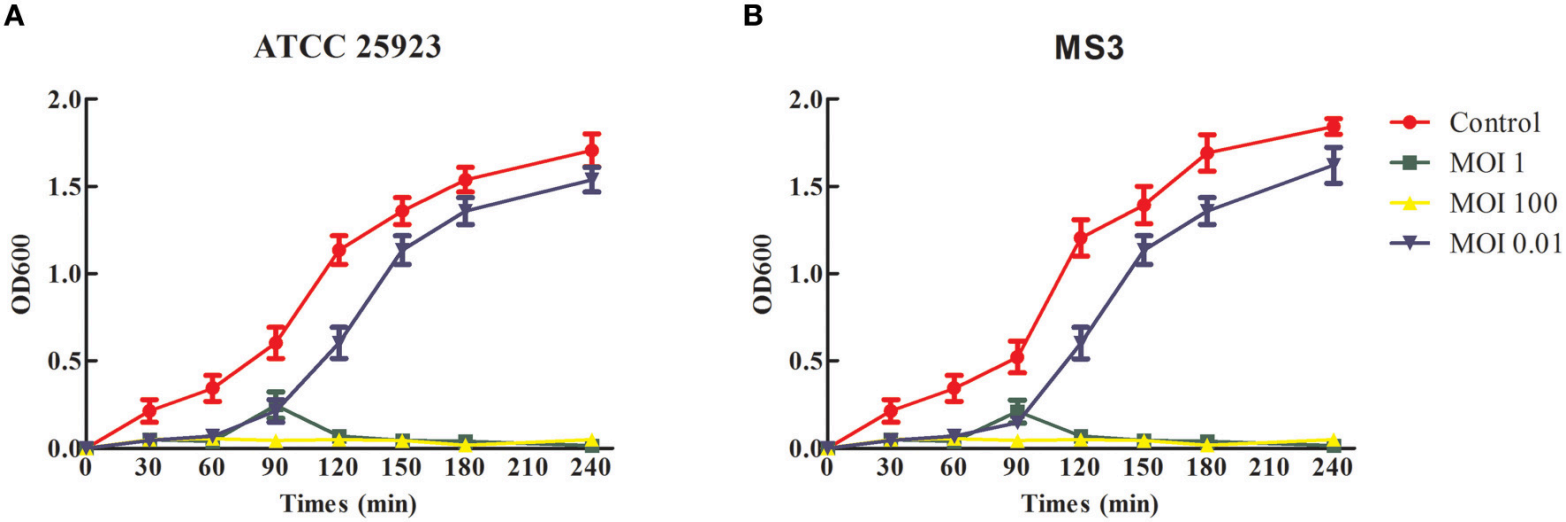
**Bacteriolytic activity of SLPW against *S. aureus in vitro***. Early exponential cultures of *S. aureus* **(A)** ATCC25923 and **(B)** MS3 strains were co-cultured with SLPW phage at MOIs of 0.01, 1, and 100, respectively. *S. aureus* cultured with a similar volume of phage diluent was used as a control. Results are shown as means ± SEM from triplicate experiments.

The phage SLPW bacteriolytic activity was tested in an early-exponential phase culture of *S. aureus* ATCC25923 and MS3 strains. The growth of these strains steadily declined at an MOI 1 and was completely inhibited at MOI 100 directly after phage administration (Figure 6). However, when the culture was administered using phage SLPW at MOI 0.01, the absorbance (OD600) continued to increase during the incubation (Figure 6). The results suggested that SLPW was highly effective against *S. aureus in vitro* and an MOI 1 was used for therapeutic study *in vivo*.

### Phage therapeutic study

Although studies indicate successful outcomes with topical phage treatment of human and animal infections involving *S. aureus*, few studies focused on the treatment of acute and lethal infection (Chhibber et al., 2013; Pincus et al., 2015). To investigate the virulence of MRSA strain MS3 in BALB/c mice, the mortality of mice was recorded daily, and followed over a period of 7 days after infection. Injection with 1 × 10^9^ and 1 × 10^7^ bacterial cells resulted in 100 and 0% death, respectively (data not shown). These doses were therefore used in subsequent experiments.

**Figure 7:**
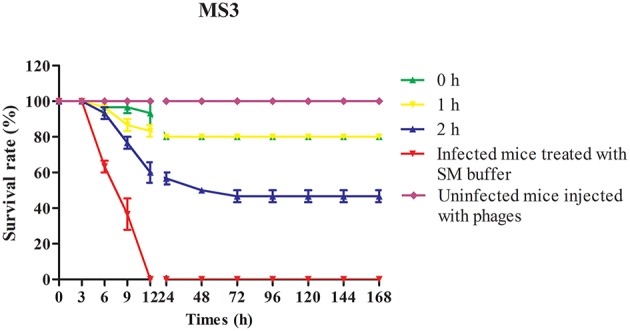
**Survival curves of mice infected with *S. aureus* MS3 strain and treated with phage SLPW**. Phage (MOIs of 1) was administered intraperitoneally into mice at 0, 1, or 2 h post-infection and the survival rates were recorded. Data shown are representative of three independent experiments using 10 mice per group, and displayed as mean ± SEM.

**Figure 8:**
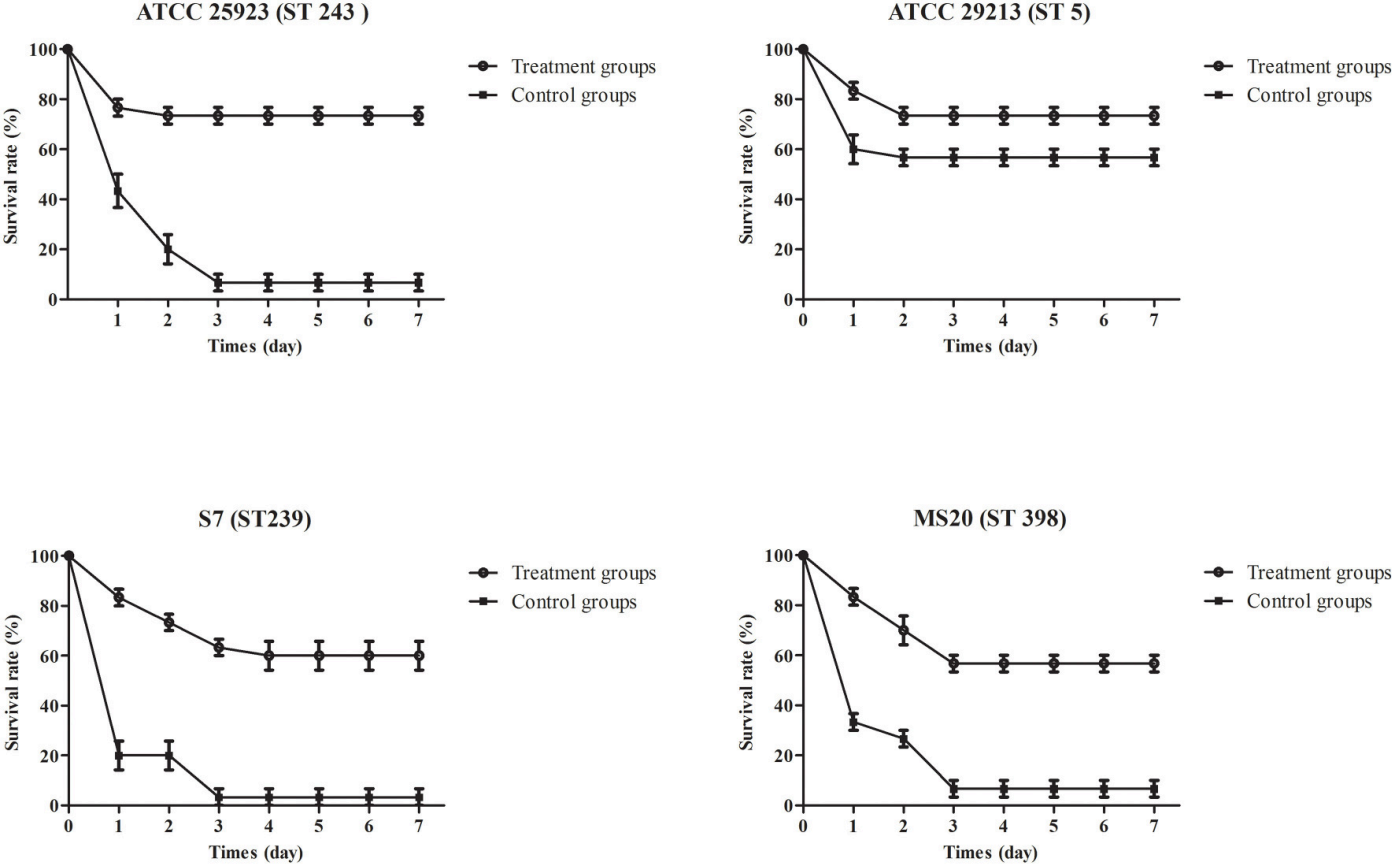
**Survival curves of mice infected with different *S. aureus* strains and treated with phage SLPW**. Phage (MOIs of 1) was injected intraperitoneally into mice at 1 h post-infection and the survival rates were recorded. Infected mice treated with SM buffer served as control. Data shown are representative of three independent experiments using 10 mice per group, and displayed as mean ± SEM.

To evaluate the therapeutic potential of phage SLPW *in vivo*, assays were performed using BALB/c mice after infection with high concentrations of *S. aureus* (1 × 10^9^ CFU/mouse). The results showed that mice treated with phage immediately (0 h) or at 1 h post-infection showed significantly higher survival rates than the control groups (infected mice treated with SM buffer) after 7 days. The survival rates following phage therapy at 0, 1, and 2 h were about 80, 80, and 50% after 7 days, respectively (Figure 7). Recent studies have shown that immediate phage treatment provided better protection than delayed administration against bacterial infection in mice (Watanabe et al., 2007; Hsieh et al., 2011). However, our study indicated that the survival rates between immediate and delayed therapy (1 h post-infection) by phage SLPW were similar (Figure 7). This finding confirmed that phage SLPW showed higher efficiency and sensitivity against *S. aureus in vivo*. In addition, phage SLPW exhibited satisfactory therapeutic effect against different sequence types of *S. aureus* following multilocus sequence typing (MLST; Figure 8).

**Figure 9:**
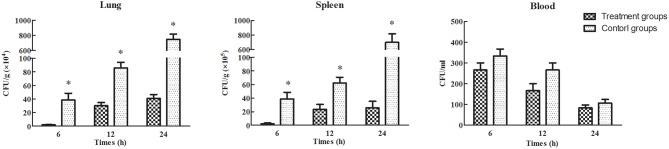
***S. aureus* concentrations in the blood and organs of mice treated with phage SLPW at 1 h post-infection**. Six mice were sacrificed from each mouse group at different time points. SM buffer was administered to infected mice, which served as control. Results are shown as means ± SEM from triplicate experiments. The Mann-Whitney U-test was used to compare the phage concentration data. Significant differences (*P* < 0.01) are indicated by asterisks.

**Figure 10:**
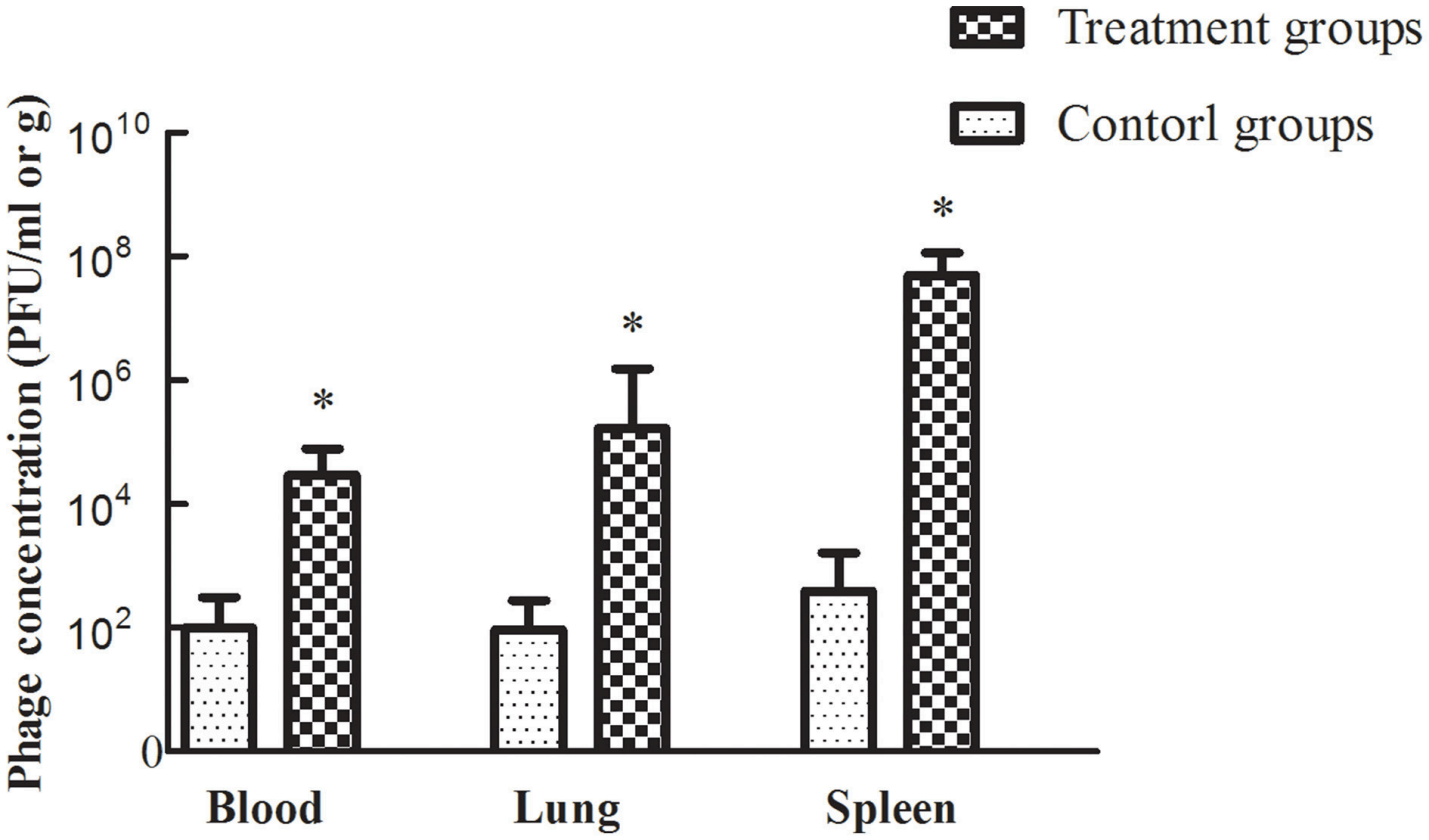
**Figure 10 Phage SLPW concentrations in the blood and organs of mice treated with phage SLPW at 1 h post-infection**. Six mice were sacrificed from each group at 24 h after the experiment. Phage was injected into uninfected mice, which served as controls. Results are shown as means ± SEM from triplicate experiments. The Mann-Whitney U-test was used to compare the phage concentration data. Significant differences (*P* < 0.01) are indicated by asterisks.

Treatment efficacy was evaluated by examining bacterial colonization in the organs and blood of mice after phage therapy (1 × 10^7^ PFU/mouse) following 1 h of infection with a non-lethal dosage of *S. aureus* (1 × 10^7^ CFU/mouse). Mice infected with *S. aureus* strains showed relatively high pathogen density in organs (Takemura-Uchiyama et al., 2014; Li et al., 2015). However, the mice treated with SLPW showed significantly lower *S. aureus* levels in the spleen and lung than the control groups at every time point (Figure 9) suggesting that SLPW was therapeutically effective against systemic infection caused by *S. aureus*. However, the *S. aureus* concentrations in the blood of the phage-administered groups were slightly lower than in the control groups (Figure 9). The results also showed that phage titers in the blood and organs at 24 h after therapy were significantly increased by nearly four orders of magnitude in the spleen and two orders of magnitude in the blood and lung compared with those in the uninfected control groups (Figure 10). Based on these results, we confirmed that SLPW contributed to resistance to *S. aureus* and enhanced mouse survival. Unfortunately, the therapeutic effect of SLPW in uninfected control mice was temporary (Figure 10) and phage therapy before infection with *S. aureus* showed no effect on survival (data not shown).

**Figure 11:**
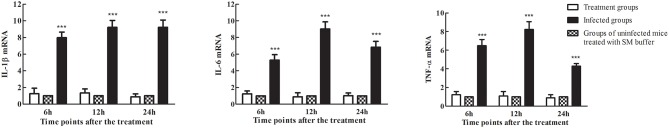
**Levels of the pro-inflammatory cytokines IL-1β, IL-6, and TNF-α in the spleens of mice treated with phage SLPW at 1 h after infection**. Six mice were sacrificed from each mouse group at different time points. SM buffer was administered to uninfected control mice. The values of pro-inflammatory cytokines in the control groups were normalized to 1.0. Levels of IL-1β, IL-6, and TNF-α mRNA were normalized to β-actin mRNA levels and were expressed as n-fold increases with respect to the control. Results are shown as means ± SEM from triplicate experiments. The Mann-Whitney U-test was used to compare the cytokine levels between infected groups and treatment groups. Significant differences (*P* < 0.001) are shown by asterisks.

The strength and efficiency of the host immune response depends on the level of pro-inflammatory cytokines including IL-1β, IL-6, and TNF-α, which are indicators of the severity of infection (Wang et al., 2014; Lee et al., 2015). The results showed that phage therapy of uninfected mice (medium-treated groups) did not alter the cytokines levels (Figure 11). Therefore, phage treatment was considered safe in mice and cytokine experiments showed no bias. The IL-1β, IL-6, and TNF-α mRNA levels were significantly lower in the phage-administered mice (treatment groups) than in untreated mice (infected groups) at every time point (Figure 11). The results suggested that phage treatment successfully attenuated inflammation caused by *S. aureus* in mice.

The adverse effects associated with bactericidal agents include the release of large amounts of pathogen-associated molecular patterns recognized by Toll-like receptors and induction of pro-inflammatory cytokines in mammals (Ginsburg, 2002; Horner and Raz, 2003). However, our studies showed that treatment of uninfected mice with phage SLPW showed no significant differences in the expression of pro-inflammatory cytokines (IL-1β, IL-6, and TNF-α) during 24 h (Figure 11). In addition, phage therapy of uninfected mice resulted in similar survival as mice injected with normal saline after 7 days (data not shown). Our findings suggest that therapy using phage SLPW was safe, although phage residues in tissues may influence normal microflora in the human body (Endersen et al., 2014). Therefore, active therapy using phage SLPW is expected to be effective in the treatment of severe systemic infection caused by *S. aureus*.

## Conclusion

In conclusion, our study investigates a lytic phage SLPW, which exhibits a wide host range, strong lytic activity and relative stability under various conditions. Rodent studies demonstrate a protective role of the phage SLPW in mice against MRSA infection, suggesting a potential antimicrobial role. Controlled clinical studies are needed to investigate the findings in animal studies.

## Author contributions

JS, YY, ZW, and PZ designed experiments; ZW and PZ carried out experiments; ZW, WJ, QF and HW analyzed experimental results; ZW and JS wrote the manuscript.

### Conflict of interest statement

The authors declare that the research was conducted in the absence of any commercial or financial relationships that could be construed as a potential conflict of interest.
