# Supplementary material for: SLPW: A Virulent Bacteriophage Targeting Methicillin-Resistant Staphylococcus aureus In vitro and In vivo
Source: Front Microbiol. 2016 Jun 15;7:934. doi: 10.3389/fmicb.2016.00934 (PMC4908117; doi:10.3389/fmicb.2016.00934)
Supplement: Supplementary file 1 [file Table1.DOCX]

Table S1 The sequence identity of the SLPW genome with other *Staphylococcus* phage

| Accession | Other phages | Phage type | Genome size (bp) | Morphology | Query cover | Ident |
| --- | --- | --- | --- | --- | --- | --- |
| AB626963.1  AY954949.1  HF937074.1 | *Staphylococcus* phage S13'  Bacteriophage 66  *Staphylococcus* phage PSa3 | Lytic  Lytic  Lytic | 18186  18199  17602 | Podoviridae  Podoviridae  Podoviridae | 97%  98%  99% | 94%  91%  91% |
| AF513033.1 | *Staphylococcus* phage phiP68 | Lytic | 18227 | Podoviridae | 98% | 91% |
| AB626962.1 | *Staphylococcus* phage S24-1 | Lytic | 18168 | Podoviridae | 93% | 93% |
| EU136189.1 | *Staphylococcus* phage SAP-2 | Lytic | 17938 | Podoviridae | 94% | 92% |
| AF513032.1 | *Staphylococcus* phage phi44AHJD | Lytic | 16784 | Podoviridae | 90% | 91% |
| KJ210330.1 | *Staphylococcus* phage GRCS | Lytic | 17869 | Podoviridae | 90% | 90% |
| JQ309827.1 | *Enterococcus* phage vB_Efae230P-4 | Lytic | 17972 | Podoviridae | 1% | 77% |
| NC007045 | *Staphylococcus* phage PT1028 | Temperate | 15603 | Unknown | 6% | 92% |
