# Supplementary material for: SLPW: A Virulent Bacteriophage Targeting Methicillin-Resistant Staphylococcus aureus In vitro and In vivo
Source: Front Microbiol. 2016 Jun 15;7:934. doi: 10.3389/fmicb.2016.00934 (PMC4908117; doi:10.3389/fmicb.2016.00934)
Supplement: Supplementary file 2 [file Table2.DOCX]

Table S2 Genome annotation of SLPW phage

| ORF | Lengths of amino acids | Function prediction or homologies | Identities of amino acids | | | | | | | | |
| --- | --- | --- | --- | --- | --- | --- | --- | --- | --- | --- | --- |
|  |  |  | S13' | 66 | PSa3 | | phiP68 | | 44AHJD | | PT1028 |
| Gp1 | 100 | Phage protein | 96% | 96% | 94% | | 98% | | 97% | | n.p. |
| Gp2 | 78 | Phage protein | 94% | 95% | 76% | | 82% | | 81% | | n.p. |
| Gp3 | 112 | Single stranded DNA-binding protein, phage-associated | 97% | 95% | 94% | | 94% | | 95% | | 32% |
| Gp4 | 59 | Phage protein | 45% | 42% | 42% | | n.p. | | 42% | | n.p. |
| Gp5 | 55 | Phage protein | 98% | 93% | 88% | | 82% | | 87% | | n.p. |
| Gp6 | 159 | Phage protein | 54% | 55% | 53% | | 54% | | 55% | | n.p. |
| Gp7 | 378 | Phage DNA packaging | 98% | 96% | 97% | | 96% | | 96% | | 26% |
| Gp8 | 755 | DNA polymerase phage-associated | 96% | 94% | 95% | | 97% | | 95% | | n.p. |
| Gp9 | 478 | Phage protein | 96% | 97% | 99% | | 97% | | 97% | | n.p. |
| Gp10 | 140 | Probable holin | 97% | 90% | 98% | | 90% | | 94% | | n.p. |
| Gp11 | 587 | Phage tail fibers | 99% | 98% | 99% | | 99% | | 99% | | n.p. |
| Gp12 | 151 | Phage tail fibers | 89% | 90% | 87% | | 91% | | 89% | | n.p. |
| Gp13 | 179 | Phage minor tail protein | 96% | 92% | 90% | | 93% | | 95% | | n.p. |
| Gp14 | 250 | Phage lysin, N-acetylmuramoyl-L-alanine amidase | 91% | 94% | 91% | | 92% | | 92% | | n.p. |
| Gp15 | 644 | Putative major teichoic acid biosynthesis protein C | 98% | 97% | 95% | | 97% | | 97% | | 28% |
| Gp16 | 251 | Phage lower collar protein | 98% | 96% | 97% | | 96% | | 96% | | 22% |
| Gp17 | 327 | Phage collar | 100% | 92% | 93% | | 92% | | 94% | | n.p. |
| Gp18 | 403 | Phage capsid and scaffold | 98% | 92% | 92% | | 93% | | 92% | | 25% |
| Gp19 | 60 | Phage protein | 98% | 98% | 97% | 98% | | 98% | | n.p. | |
| Gp20 | 133 | Phage protein | 81% | 71% | 69% | 64% | | 65% | | n.p. | |
|  |  |  |  |  |  |  |  |  |  |  |  |

n. p. gene not present
