# Supplementary figures and images for: SLPW: A Virulent Bacteriophage Targeting Methicillin-Resistant Staphylococcus aureus In vitro and In vivo
Source: Front Microbiol. 2016 Jun 15;7:934. doi: 10.3389/fmicb.2016.00934 (PMC4908117; doi:10.3389/fmicb.2016.00934)

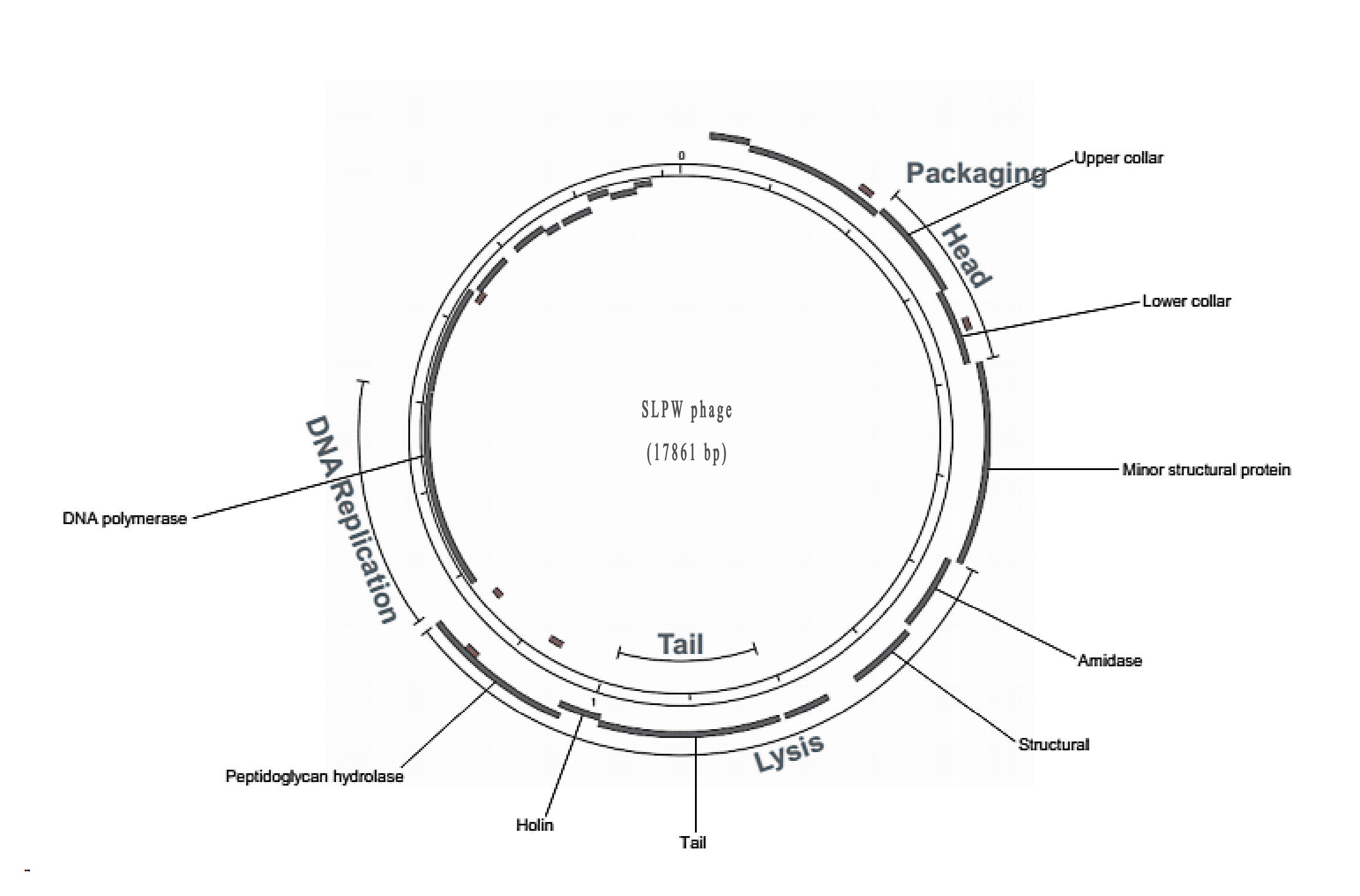

Supplement: Supplementary file 3 [file Image1.TIF]
